# Laying low: Rugged lowland rainforest preferred by feral cats in the Australian Wet Tropics

**DOI:** 10.1002/ece3.9105

**Published:** 2022-07-13

**Authors:** Tom Bruce, Stephen E. Williams, Rajan Amin, Felicity L'Hotellier, Ben T. Hirsch

**Affiliations:** ^1^ Centre for Tropical Environmental and Sustainability Science College of Science and Engineering James Cook University Townsville Queensland Australia; ^2^ Zoological Society of London London UK; ^3^ Australian Wildlife Conservancy Subiaco East Western Australia Australia; ^4^ Smithsonian Tropical Research Institute Panama Panama

**Keywords:** camera‐trap, elevation, Felis catus, national park, occupancy, topography

## Abstract

Invasive mesopredators are responsible for the decline of many species of native mammals worldwide. Feral cats have been causally linked to multiple extinctions of Australian mammals since European colonization. While feral cats are found throughout Australia, most research has been undertaken in arid habitats, thus there is a limited understanding of feral cat distribution, abundance, and ecology in Australian tropical rainforests. We carried out camera‐trapping surveys at 108 locations across seven study sites, spanning 200 km in the Australian Wet Tropics. Single‐species occupancy analysis was implemented to investigate how environmental factors influence feral cat distribution. Feral cats were detected at a rate of 5.09 photographs/100 days, 11 times higher than previously recorded in the Australian Wet Tropics. The main environmental factors influencing feral cat occupancy were a positive association with terrain ruggedness, a negative association with elevation, and a higher affinity for rainforest than eucalypt forest. These findings were consistent with other studies on feral cat ecology but differed from similar surveys in Australia. Increasingly harsh and consistently wet weather conditions at higher elevations, and improved shelter in topographically complex habitats may drive cat preference for lowland rainforest. Feral cats were positively associated with roads, supporting the theory that roads facilitate access and colonization of feral cats within more remote parts of the rainforest. Higher elevation rainforests with no roads could act as refugia for native prey species within the critical weight range. Regular monitoring of existing roads should be implemented to monitor feral cats, and new linear infrastructure should be limited to prevent encroachment into these areas. This is pertinent as climate change modeling suggests that habitats at higher elevations will become similar to lower elevations, potentially making the environment more suitable for feral cat populations.

## Graphical abstract

Feral cats are responsible for the decline of multiple native species both globally and in Australia. However, we know very little about their ecology in tropical rainforests. This study demonstrated despite predictions of cats being absent from wet complex habitats, feral cats were present in all national parks surveyed and prefer low elevation, rugged terrain in rainforests.

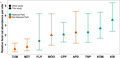

## 1 INTRODUCTION

Invasive species are a major threat to global biodiversity (Bellard et al., 2016). Invasive mammalian predators are thought to cause substantial declines in prey species (Doherty et al., 2016). Recent meta‐analyses demonstrate that 45% of endangered and critically endangered species globally are threatened by invasive mammals, both from predation, and indirect effects such as competition for resources (Dueñas et al., 2021).

The impacts of invasive vertebrate predators are of particular concern within Australia, where many native prey have not evolved with placental mammalian predators (Moseby et al., 2016). The negative effects of invasive predators can be exacerbated by environmental factors, such as habitat fragmentation and altered fire regimes (Doherty, Davis, et al., 2015). Since European colonization 33 mammalian species have gone extinct in Australia (Hohnen et al., 2020). Twenty‐two and 13 of these extinctions have been causally linked to feral cats (*Felis catus*) and red foxes (*Vulpes vulpes*), respectively (Woinarski et al., 2015). It is worth noting that experimental evidence directly demonstrating extinctions of endemic fauna driven by invasive predators is currently limited, particularly in Northern Australia (Preece & Fitzsimons, 2022).

The Australian Wet Tropics (AWT) is the largest remnant tropical rainforest in Australia. Despite accounting for only 0.12% of Australia's landmass, it is Australia's most species‐rich and diverse area (Williams et al., 2009). The AWT contains approximately 30% of Australian mammal species, and 90 vertebrate species are endemic to the region (Williams, Vanderwal, et al., 2010; WTMA, 2016). Historically, the biggest threats to rainforest species were habitat loss and fragmentation due to land clearing (Roberts et al., 2021). Currently, climate change and invasive species are being viewed as the primary threats to the AWT (Williams et al., 2003; WTMA, 2020). These threats can act synergistically to impact native habitat and species: for example, fragmented forest patches are more vulnerable to increased penetration by invasive species, altered climates, and increased aridity due to edge effects subsequently drive more frequent and damaging bush fires (Almeida et al., 2019; Laurance & Williamson, 2001; Olson et al., 2006). Another conservation threat that can exacerbate the previously mentioned issues is the presence of roads (Laurance et al., 2015). Roads may accelerate the spread of invasive vertebrate predators by allowing species to move efficiently across landscapes and colonize areas that they would otherwise take longer to reach (Hradsky et al., 2017; Raiter et al., 2018). Studies in Australia have generally found feral cats preferentially travel along linear features like roads but see Bridges et al. (2015) (Wang & Fisher, 2012; Wysong, Hradsky, et al., 2020; Wysong, Iacona, et al., 2020). Within the AWT there is an extensive network of roads bisecting otherwise intact habitats, and feral cats may have been using them undetected for decades.

Feral cats are regarded as one of the most destructive invasive mesopredators to arrive in Australia (Murphy et al., 2019; Woinarski et al., 2017, 2018). Even at low densities, feral cats can substantially impact prey species populations. The impacts of feral cats in tropical rainforests are poorly understood globally, with most studies occurring on islands, such as Madagascar, Hawaii, and other Pacific islands (Farris et al., 2016; Lavery et al., 2020). Studies on feral cat ecology within Australia are more prevalent in arid and open areas. The regional bias in surveys is most likely due to animals being easier to detect in open, arid habitats than in dense and complex forests (Anderson et al., 2015; Trolle et al., 2008). Lower detection rates of feral cats, because of closed habitat structure and their cryptic behavior, are thought to have caused underestimations of feral cat abundance in forest environments (Denny & Dickman, 2010). This has led some studies to suggest that rainforests potentially support lower densities of feral cats than more open habitat types, resulting in neglected conservation planning for feral cat management in tropical forests (Dickman, 1996; Legge et al., 2017).

Feral cat habitat preference studies in Australia often conflict with global findings, thus predicting feral cat presence and abundance in Australia is not straightforward (Doherty et al., 2014; Rees et al., 2019). In a global review of 27 studies on feral cat habitat preferences, Doherty et al. (2014) found a preference for more complex habitats characterized by heterogeneous understory vegetation divided by linear features, such as roads or riparian corridors. They recommend targeting these structures for feral cat monitoring. In contrast, recent studies within Australia indicated that complex habitats, as measured by understory vegetation and topographic complexity, are avoided by feral cats due to an assumed reduction in hunting success (Hohnen et al., 2016; McDonald et al., 2020; Stobo‐Wilson et al., 2020). These contradictory results justify further efforts to identify the predictors of feral cat distributions and relative abundance in the AWT.

Here, we investigate spatial and ecological patterns of feral cat abundance and occupancy within the AWT. Given previous studies on feral cat ecology, we test whether:

1. Feral cat occupancy will be higher in rainforests than in eucalypt forests (Rees et al., 2019).
2. There is a positive relationship between habitat complexity and feral cat occupancy (Doherty et al., 2014).
3. Feral cats will be detected more at camera‐traps placed facing roads than those in the forest (Wysong, Iacona, et al., 2020).

## 2 METHODS

### 2.1 Study area

**FIGURE 1:**
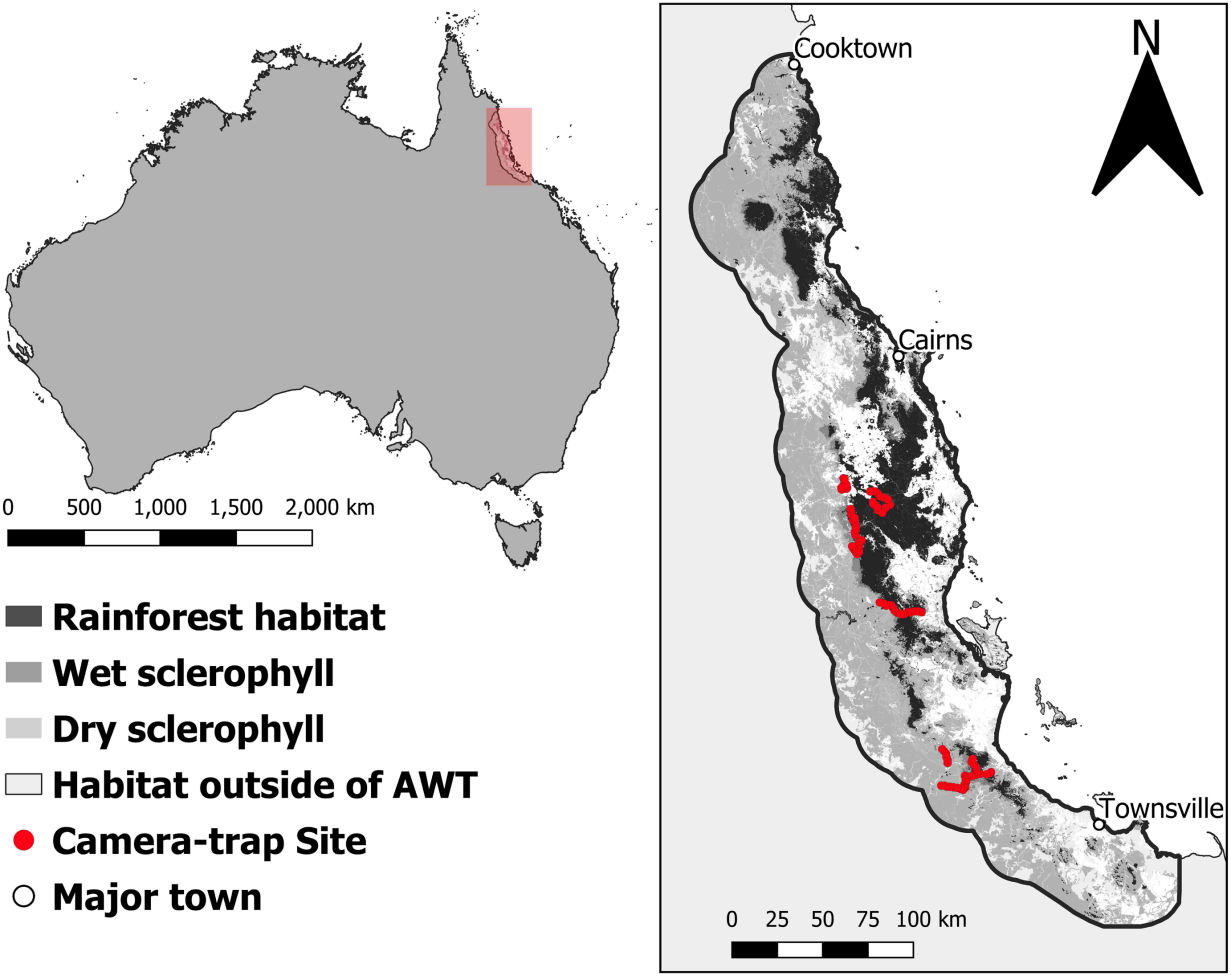
Location of Australian Wet Tropics within Australia, displaying broad habitat types and camera locations

The AWT is a World Heritage Site covering approximately 894,420 ha in northeast Queensland, Australia. There are multiple national parks and protected forest reserves in the region (Pert et al., 2012). Habitat within the AWT is composed of a patchwork of rainforest, and open wet and dry sclerophyll forest. The area is surrounded by anthropogenically disturbed lowlands that include urban populations, cattle pastures, and plantations such as sugarcane (Figure 1; Morrant, Wurster, et al., 2017). There are two distinct seasons within the region. The wet season, during which over 60% of the annual rainfall occurs (December–March) and the dry season (April–December; Goosem, 2002).

The heterogeneous nature of the terrain and environment within the AWT promotes a diverse range of climatic conditions over relatively small spatial scales (Williams, Shoo, et al., 2010; Williams, Vanderwal, et al., 2010). The altitudinal profile ranges from sea level to a maximum of 1622 m a.s.l. Elevation influences both rainfall patterns and temperature, with higher elevation mountain tops experiencing lower temperatures (minimum 5°C) and higher rainfall (12,000 mm annually) compared with the lowlands, which are hotter (maximum 35°C) but still humid, with an average of 1300 mm of rain annually (Department of Environment and Science (DES), 2019; Nix & Switzer, 1991).

### 2.2 Study design

By placing camera‐traps in a range of environments across an elevation gradient, we aimed to identify predictors of feral cat habitat use and abundance in the Wet Tropics. Our sampling was designed to examine feral cat habitat use between the following environments: rainforest versus more open eucalypt forests and complex versus simpler forest structures. Camera‐trap surveys were carried out in four national parks, one state forest, one cattle property, and one wildlife sanctuary (Table 1).

**TABLE 1:** Camera‐trap survey site locations and details for occupancy and relative abundance, for feral cats in the Australian Wet Tropics

Property	Designation	Size km^2^	No. sites	Survey start date	Survey end date	Effort days active	Feral cat independent detections	Average elevation at camera‐trap site (min–max)
Wooroonooran	National Park	314	18	16/04/2019	30/05/2019	1383	102	648 (483–819)
Koombooloomba & Tully falls	National Park	463	20	06/09/2019	23/10/2019	1195	108	789 (696–981)
Paluma	National Park	794	18	05/11/2019	29/01/2020	2608	2	803 (502–943)
Cattle Property Paluma	Cattle property	210	6	07/11/2019	05/02/2020	618	53	734 (699–771)
Kirrama	National Park	175	18	19/07/2019	05/09/2019	1616	204	572 (164–763)
Tumoulin	State forest	19	17	12/04/2019	28/05/2019	1451	26	1006 (916–1061)
Mount Zero‐Taravale	Wildlife Sanctuary	591	13	07/05/2020	23/07/2020	1415	29	777 (587–933)
						10,286	524	

One hundred and eight Camera‐trap pairs were placed along main roads, four‐wheel‐drive tracks, and walking trails with one camera‐trap facing into the road and another camera‐trap positioned in the habitat, 50 m perpendicular from the road. The camera was placed in the habitat to test the hypothesis feral cats would be more likely to use roads in tropical forests. A length of 50 m was used to counteract potential spatial avoidance by feral cats of habitat features favored by dingoes (*Canis lupus dingo*), the apex predator in Australia (Fancourt et al., 2019). We treated both cameras, on‐road and off‐road, as a single site for analyses due to their proximity to one another. Each camera‐trap pair's planned location was spaced 2.2 km along the road; this distance exceeded the predicted home range of 1.16 km for feral cats in productive, low seasonality environments like rainforests and matched the home‐range estimate of female feral cats in a montane rainforest in Hawaii (Bengsen et al., 2016; Smucker et al., 2000). Nineteen opportunistic camera‐trap pairs were placed on walking trails and old roads that were not present on maps of the study areas. These opportunistic deployments were placed 500 m–1.98 km from the nearest camera‐trap pair using the road. This deployment strategy resulted in an average distance of 1.81 km between camera‐trap sites based on the distance to the nearest neighboring camera‐trap site using the road network. Due to uncertainty around cat home ranges in the study area, there is a potential for individual feral cats to be detected at multiple cameras. To be conservative, we interpret the occupancy results as the probability of site use rather than true occupancy, which is the same approach as other studies on feral cats in Australia and carnivore surveys generally (Doherty et al., 2021; MacKenzie et al., 2017; Rogan et al., 2019). Surveys were conducted between April 2019 and July 2020 for a minimum of 6 weeks per survey. We did not use baits or lures in front of the camera‐traps, as their use can influence species behavior in an unknown way if it has not been evaluated previously, causing either a repellent, attractive, or neutral response (Mills et al., 2019; Rocha et al., 2016).

### 2.3 Camera placement and settings

We followed the general guidelines for camera‐trap deployment recommended by Meek et al. (2012). At each camera‐trap site, a Bushnell Trophy Aggressor No‐glow camera (Bushnell Outdoor Products) was placed facing the road within 200 m of the planned point. Camera‐traps were placed facing the road, perpendicular to the direction animals would travel, to maximize the likelihood of detecting our target species (Wang et al., 2019). Camera‐traps were positioned at a height of 20–45 cm from the ground, as this is the approximate height of the center of mass for an adult feral cat (McGregor, Legge, Jones, et al., 2015). Cameras were angled to be parallel with the terrain they were facing. Cameras were not orientated at a specific compass bearing as we were following the road to place cameras and prioritized angling the camera perpendicular to the animal's expected travel route. For each road camera location, a single No‐glow Bushnell Natureview (Bushnell Outdoor Products) was placed 50 m from the road in the forest with a consistent and unobstructed field of view. Vegetation and debris were removed to a minimum distance of 4 m in front of the camera‐trap where necessary to ensure a clear field of vision over the survey period. All camera‐traps were programmed to take three images per trigger, with no delay between triggers (recovery time between consecutive photos = 0.62 s for Bushnell Aggressor, 0.7 s Bushnell Natureview). Due to equipment failure, four of the forest camera‐traps at Mount Zero‐Taravale Wildlife Sanctuary were replaced with Reconyx Hyperfire PC800 infrared cameras. While different camera‐trap brands have different detection probabilities, the fact that feral cats were rarely detected off‐roads in our study makes it unlikely this difference affected our results (Palencia et al., 2022). The infrared flashes on the road camera‐traps were set to high to ensure as much of the road as possible would be visible in the pictures, while forest infrared flashes were set to low to avoid overexposing the image. The remaining settings were all left at their factory defaults.

### 2.4 Predictor variables of feral cat occupancy

To investigate spatial factors that influence feral cat occupancy, we collated environmental and anthropogenic variables that are hypothesized to alter feral cat distribution and site use (Doherty, Dickman, et al., 2015). We tested hypotheses related to terrain (elevation, terrain ruggedness index), habitat (habitat type, understory vegetation density), anthropogenic disturbance (distance from the nearest human population, forest integrity, and habitat fragmentation), primary productivity (mean annual rainfall), prey populations (biomass index of prey species), invasive herbivores (invasive herbivore trapping rate), and altered fire regimes (fire regime intensity). For a complete list of the variables considered, how they were calculated, and the hypothesis they are linked see Table S1. As there is little known about the spatial scale at which predictor variables affect feral cat occupancy, each model covariate value was averaged over a 2.2 km^2^ circular area around the camera point (Stobo‐Wilson et al., 2020). The averaging of a spatial covariate was done to capture the variable at a scale relevant to the home range of a feral cat in rainforest habitats. This is pertinent as the effective area surveyed by the camera‐trap is only relative to the sensor's detection zone, not the wider area we averaged for the environmental covariates (MacKenzie et al., 2017). All continuous variables were checked for correlation before analyses. If any variable pairing resulted in a Spearman correlation coefficient >0.7, we only retained what we considered to be the most biologically relevant variable. The remaining variables were then scaled to improve model performance. To consider survey biases, we used observation level covariates in the analysis. We used the survey effort (the total number of days both cameras were actively trapping at each site for each occasion) to account for variable camera performance of both the road and off‐road cameras at each camera‐trap site influencing detection probability.

## 3 ANALYSIS

Camera‐trap images were imported into Timelapse2 software (Greenberg & Godin, 2012). Images were classified and tagged with data on the cause of the camera trigger and the contents of the image (blank, species, human, vegetation movement, fire, etc.). A trapping rate was calculated for feral cats by dividing the number of independent photographic events, defined as any successive captures separated by >60 min, by the number of days the camera was operating for and then multiplying by 100 (Rovero & Marshall, 2004). We investigated the effect of environmental and anthropogenic covariates on feral cat occupancy by jointly modeling occupancy and detection probability using single‐season, single‐species Royle Nichols (RN) occupancy modeling, a hierarchical framework that considers imperfect detection of species (MacKenzie et al., 2002; Royle & Nichols, 2003). The RN occupancy model generates two parameters, the abundance per sampling unit (λ), by considering the positive relationship between the abundance of a species and the resultant heterogeneity in detection probability (*r*), the probability of detecting one individual at a site. The main assumption is that at sites with more individuals present, there will be a higher detection probability. We derived site occupancy (Ψ) from λ to understand how covariates affected patterns of feral cat occupancy. Importantly, the RN model allows an estimate of site‐specific occupancy and a measure of relative abundance of feral cats across our different study areas. Unlike a basic occupancy model, the RN model considers that heterogeneity in detection probability can be partially explained by variation in abundance between camera‐trap sites (Sollmann, 2018). As our studies were carried out in different national parks, we feel it is reasonable to assume that differences in abundance of feral cats at different sites is likely to affect detection probability, making the RN model more appropriate than a traditional single‐season occupancy model. By using a RN approach, we calculate the mean camera‐trap site‐specific relative abundance estimates. In this case, we estimate the mean number of feral cats per camera‐trap site^−1^ in each study area, to match other studies conducted in Australia (Fancourt et al., 2021; Taggart et al., 2019).

We generated detection histories using independent captures of feral cats in the R package “CamtrapR” (Niedballa et al., 2016). A five‐day period was used as a sampling occasion. A sampling occasion was included in the analysis if either camera was operational for 3 days or more. We define a site, as a location where we deployed a road and an off‐road camera‐trap, and a study area as the broad landscape the sites were deployed in, for example, each individual national park or state forest. Occupancy analysis was undertaken in R package “unmarked” (Fiske & Chandler, 2011). Paluma national park was excluded from analysis as only two feral cats were detected during 2608 camera‐trap days. This extremely low level of detections violate the minimum number of detections needed to conduct these analyses at Paluma national park.

We utilized a widely used approach to model fitting and selection within the occupancy and camera‐trapping literature (Linkie et al., 2007; Rovero et al., 2014). Firstly, a null model was developed by holding detectability and occupancy constant *r*(.) λ(.). We then built models in a stepwise manner. We established the best detection model by holding occupancy constant and allowing detectability to vary with covariates *r*(covariate) λ(.). The best performing model was selected based on the Akaike information criterion corrected for small sample sizes (AICc) using the R package AICcmodavg (Mazerolle, 2020), whereby any models with a ΔAICc score of <4 were plausible hypotheses for describing the data (Burnham et al., 2011). If multiple models were within four ΔAICc values, then the most parsimonious model was selected. The detection covariate that had the most support was taken forward to estimate the drivers of site occupancy.

Subsets of covariates predicting site occupancy were modeled following the hypotheses stated in Table S1. All combinations including univariate and multivariate models were run within a hypothesis *r*(covariate) λ(covariate). The null model *r*(.) λ(.) and detection model *r*(covariate) λ(.) were also included to compare if site occupancy models had more support than the null. The top model for each hypothesis, <4 ΔAIC from the next most supported model, was regarded as potentially important for predicting site occupancy and taken forward to the final comparison of the best performing models from all hypotheses. If multiple models were within four ΔAICc values, then any models within this threshold were taken forward for the final selection. We then used the full suite of any models that were assessed to have evidence of support according to AICc in a final model comparison of potential hypothesis of feral cat occupancy in the AWT. Using the “evidence” function of AICcmodavg, evidence ratios were then used to infer which alternative hypothesis had the most support according to the data (Burnham et al., 2011). AICcWt was also used to describe the weight of evidence of the model in question being the most supported among the other candidate models (Burnham & Anderson, 2002). To plot the effect of covariates on occupancy probability, we transformed the Royle Nichols Poisson mean of λ to probability of site use (Ψ), using the “lambda2psi” function in the “unmarked” package. When displaying the effect of a single predictor variable on occupancy, we held the other variable at its mean value to assess the effect of a single variable on occupancy.

A global model was generated using all the variables considered for both the occupancy and detection components of the model. The c‐hat and chi‐squared values used to assess model dispersion were generated using the MacKenzie and Bailey (2004) goodness of fit test, conducted using 10,000 simulations for the global model (Table S3). If the global model had a c‐hat >2, it would be rejected as overdispersed, suggesting heterogeneity in the data is explained by a variable not yet included in the model (Farris et al., 2016; MacKenzie & Bailey, 2004).

## 4 RESULTS

In total, 10,286 days of survey effort were carried out, and 372,475 images were taken. Of these images, 119,330 were classified as images of wildlife and 2540 of these images were of feral cats. A breakdown of the number of camera‐trap days per area and other related figures is presented in Table 1.

**FIGURE 2:**
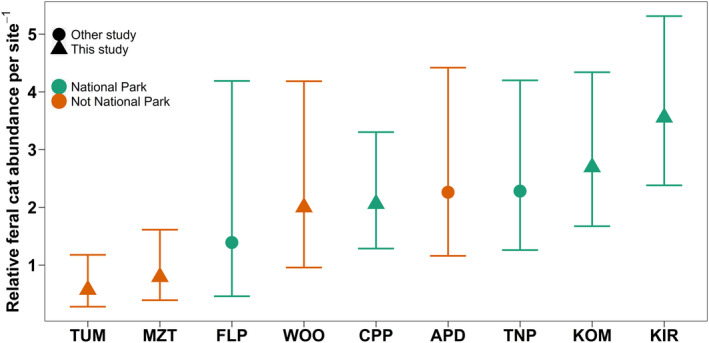
Relative abundance of feral cats per site, error bars are 95% confidence intervals. Green points represent sites that are designated as national parks, while orange points represent sites of other designations. The circles are from sites that were surveyed in this study and triangles are data points from other published studies. APD, agricultural property dingo; CPP, cattle property Paluma; FLP, Fleurieu peninsula; KIR, Kirrama National Park; KOM, Koombooloomba National Park; MZT, mount zero‐Taravale wildlife sanctuary; TNP, Taunton National Park; TUM, tumoulin forestry reserve; WOO, Wooroonooran National Park

Feral cat relative abundance in the AWT was higher than, or similar to other studies that used a similar survey and analysis approach in Queensland and South Australia (Fancourt et al., 2021; Taggart et al., 2019). National parks generally had higher relative abundance estimates than non‐national park areas (Figure 2). There were 524 independent detections of feral cats throughout the duration of the surveys, resulting in a trapping rate of 5.09 photographs/100 days, approximately 11 times higher than the only other estimate for the region of 0.45 photographs/100 days (recalculated from Table 1 [Rowland et al., 2020]). Feral cats were detected at least once at 63/90 sites included in the occupancy analysis. When comparing sites within the AWT, the relative abundance of feral cats was higher in Kirrama NP than Mount Zero‐Taravale Wildlife Sanctuary and Tumoulin Forest Reserve (Figure 2). Kirrama NP, Koomboloomba NP, and the Cattle Property all had similar relative abundances, as all the 95% confidence intervals overlapped (Figure 2).

**FIGURE 3:**
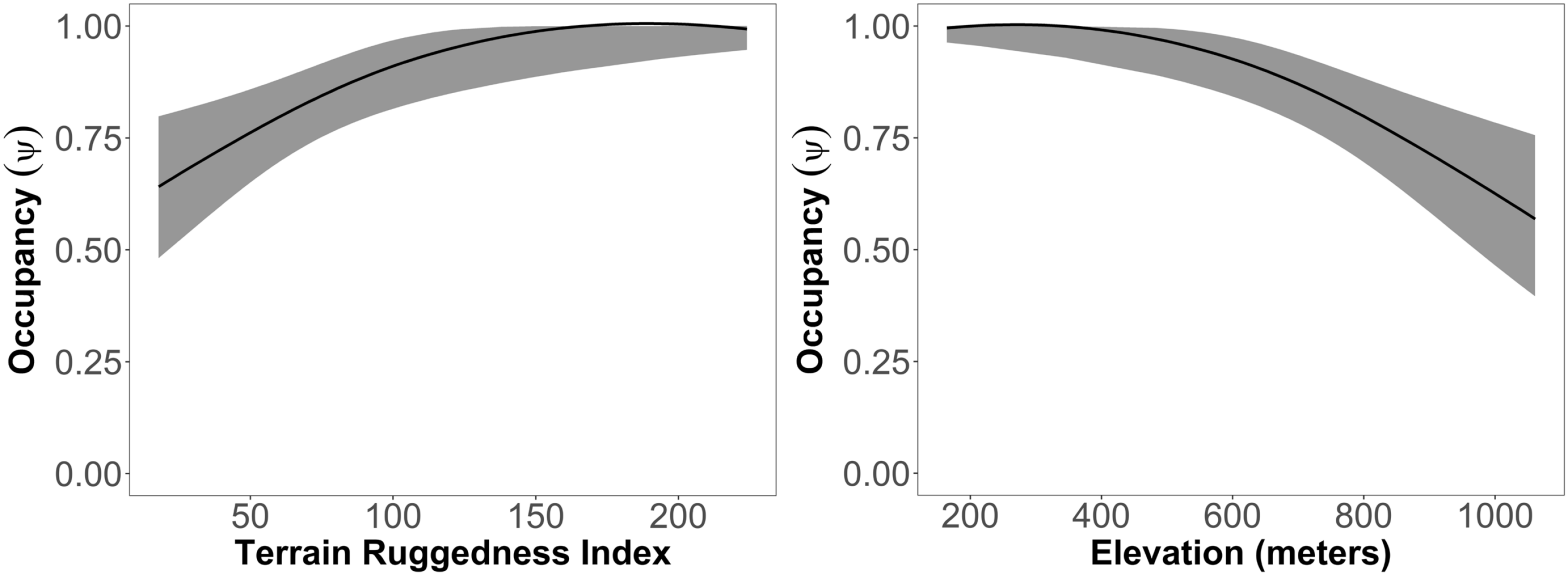
Plot showing the occupancy components of the top selected model, based on the chosen covariates. The effect of terrain ruggedness and elevation on the probability of feral cat site use (Ψ) with the other covariate held at its mean value due to the model being synergistic, ±95% confidence intervals

Feral cats preferred lower to mid‐elevation areas with more rugged terrain in the surrounding habitat (Figure 3). They were more likely to be detected with increasing survey effort at a camera‐trap site. The most parsimonious model with the highest support was a synergistic interaction (*β* = 0.31) between elevation (*β* = −0.43) and terrain ruggedness (*β* = 0.41). This means the combined effect of elevation and ruggedness was greater than a simple additive relationship. Detection was best described with increasing total effort (*β* = 0.43; Table 2) with more camera‐trap days per station per occasion, improving detection probabilities. The probability of site use by feral cats had strong evidence that it was higher in rainforest habitats than in eucalypt forests (*β*‐Rainforest = 0.85; Figure S1). Habitat type was the second most supported model when compared with all the final models, with a ΔAICc of 2.45 (Table S2). The top two models, elevation and terrain ruggedness, and habitat type using AICcWt, explained 93% of the predictive power contained in the models (Table S2). According to evidence ratios, the top model of *r*(Camera‐trap effort) λ(Elevation × Terrain ruggedness index) had approximately three times as much support compared with the second‐best model *r*(Camera‐trap effort) λ(Habitat type); (Evidence ratio: 3.4).

**TABLE 2:** Beta coefficients and standard errors for feral cat occupancy and detection probability (*p*) which have been derived from the most supported model according to ΔAICc using a single‐season Royle Nichols abundance model $r\;\left(\text{Camera}-\text{trap effort}\right)\;\lambda \left(\text{Elevation}*\text{Terrain ruggedness index}\right)\;$

Parameter	*β* estimate	*SE*	0.25	0.975
*r* (Intercept)	−1.21	0.15	−1.52	−0.91
*r* (Camera‐trap effort)	0.43	0.10	0.23	0.64
λ (Intercept)	0.59	0.15	0.30	0.87
λ (Elevation)	−0.43	0.12	−0.67	−0.19
λ (Terrain ruggedness index)	0.41	0.14	0.14	0.68
λ (Elevation* Terrain ruggedness index)	0.31	0.10	0.12	0.50

## 5 DISCUSSION

This study is the first systematic study of feral cats in the AWT region. We found that feral cats are distributed throughout the AWT and are more abundant than previously thought. An important distinction between this study and others is that we have taken into account detection probabilities, which make these findings significantly more robust, and provides a baseline for comparison across time and different locations (Burton et al., 2012; Davies et al., 2018; Hayward & Marlow, 2014).

Previous studies have suggested that feral cats could be found in very low densities in the AWT. This suggestion was based on the continental scale feral cat density model, which has been used to infer how many invertebrates, reptiles, birds, amphibians, and small mammals are consumed by cats annually (Murphy et al., 2019; Woinarski et al., 2017, 2018, 2020; Woolley et al., 2020). Cat density patterns in the continental models are probably accurate for semi‐arid and arid Australia, due to multiple existing density estimates from these environments. Future revisions of the models, and subsequent predictions, could be updated with contemporary estimates of cat density from wet and rugged forest habitats. This would augment their relevance and predictive power across a wider range of habitats in Australia. When we compared our relative abundance estimates to other studies within Queensland and South Australia, AWT sites had similar or higher relative abundance (Fancourt et al., 2021; Taggart et al., 2019). We did not include the estimates from Kangaroo Island in Taggart et al. (2019), as the drivers of feral cat relative abundance and distribution are likely to differ from those on the mainland. Similar results have been found in other wet complex forest habitats, such as the Otways in Victoria, where feral cat densities were five times higher than predicted (Rees et al., 2019). These results highlight the need for eco‐region‐specific assessments when considering feral cat distribution and relative abundance.

The most important factors for predicting feral cat site use in the AWT were elevation and topographic complexity. Elevation drives many environmental patterns and processes in our study areas, with extensively reported effects on species distributions and community structure (Nowrouzi et al., 2016; Wardhaugh et al., 2018; Williams, Shoo, et al., 2010). Feral cats are no exception to this: their occupancy declined with increasing elevation. Other studies have demonstrated that feral cats prefer lower elevation sites (Recio et al., 2014). The ecological reasons behind this relationship remain unclear. However, precipitation and prey abundance are thought to be vital factors explaining feral cat distribution, and elevation plays a significant role in both variables. High elevation sites within the AWT are consistently wet throughout the year, and a process known as cloud stripping provides significant water input into the system even during the dry season (McJannet et al., 2007). Cloud stripping occurs when montane forests intercept clouds, causing condensation to form on plant surfaces due to their large surface area. The increased condensation creates a high dew point and causes the air to be consistently saturated, driving species distributions within montane ecosystems (Nowrouzi et al., 2016; Olson, 1994). As feral cats are known to move less with increasing rainfall, the continually moist conditions at higher elevations could cause the environment to become less tolerable for feral cats (Coughlin & Van Heezik, 2014).

We suggest that prey availability is unlikely to be a critical factor in explaining the relationship between elevation, occupancy, and relative abundance of feral cat populations in the AWT. The peaks of species richness and abundance are distributed relatively evenly across the elevation gradient for small mammals (S.E. Williams, unpublished data), which provides ample prey for feral cats regardless of elevation. As small mammals constitute 70% of the diet of feral cats in the region (Rowland et al., 2020) and feral cat population densities and their degree of home‐range overlap are primarily driven by prey abundance (Edwards et al., 2001), one would expect relative abundance to be uniform across the elevation gradient and thus occupancy to remain similar if prey availability were responsible for this pattern. Consequently, the accelerated decline of occupancy for feral cats after mid‐elevation is more likely due to other factors, such as increasingly harsh environmental conditions in terms of rainfall and temperature. This relationship contrasts with other studies in Australia, where prey densities were a critical determinant of feral cat abundance and distribution (Greenville et al., 2014; Letnic & Dickman, 2010). However, it is notable that occupancy declined from 1 (95% CI = 0.96–1) at 164 m to 0.57 (95% CI = 0.40–0.76) at 1061 m, meaning that feral cats are still likely to use habitat at higher elevations.

This study found a preference for topographically complex habitats. The topographic ruggedness index describes how much elevation varies, with higher values associated with more rugged terrain. In the context of the AWT, higher ruggedness values are likely indicative of boulder fields and steep drop‐offs in the environment. Most previous studies in Australia concluded that feral cats avoid topographically complex areas due to a reduction in hunting success in more structurally complex habitats (Hohnen et al., 2016; McDonald et al., 2017; McGregor, Legge, Potts, et al., 2015; McGregor et al., 2014). Due to sparse literature on feral cat ecology in rainforests, we can only hypothesize why feral cats in our study system might prefer more topographically complex terrain. It is feasible that predator avoidance and shelter may explain a preference for topographically complex habitat. Doherty et al. (2014) proposed a hierarchy of factors driving feral cat habitat use, with predator avoidance, prey availability, human resource subsidies, and shelter being the most critical determinants of habitat use. In our study, topographic complexity might provide protection from dingoes, as dingoes are more effective hunters in flatter areas (Morrant, Johnson, et al., 2017; Stobo‐Wilson et al., 2020). Feral cats in subalpine forests in New Zealand have been shown to require shelter in forests due to wet and cold conditions, and despite the AWT having more rainfall, the temperature in the uplands is similar to the summer temperatures in the New Zealand study (Harper, 2007). Even though it was conducted in a temperate forest, the other study demonstrates that feral cats need permanent shelter from rainfall. Areas of higher topographic complexity in rainforest habitat may provide greater availability of shelter. In addition, studies of Northern quolls (*Dasyurus hallucatus*) in Australia (native carnivores similar in size and ecology to feral cats) have shown that rocky and therefore topographically complex areas can provide reliable shelter from harsh weather reviewed by Moore et al. (2021).

Feral cat occupancy was much higher in rainforest habitats than in eucalypt forests. A lack of small mammal declines in wet and rugged habitats has been invoked to imply that feral cats likely occur in lower abundance in rainforests (Murphy et al., 2019; Radford et al., 2018). Our results, along with those of Rees et al. (2019), challenge the idea that native fauna residing in mesic forests in Australia are less likely to be exposed to feral cat populations.

Roads are known to facilitate access for invasive mammalian predators (Goosem, 2007; Laurance et al., 2009; Raiter et al., 2018). Our study highlights the potential influence roads have in the AWT regarding invasive species. Out of 524 feral cat records, 14 were from cameras placed in the forest away from the road. There is the potential that the wider field of view of the road camera compared with the forest camera could be responsible for the increase in feral cat detections. We feel that our conclusion that feral cats prefer to use roads is well supported and has been found by other studies in Australia (Dawson et al., 2018; Wysong, Iacona, et al., 2020). By preferentially using roads, feral cats can improve their foraging efficiency in complex habitats by using habitat edges formed by the roads specifically for hunting. Edge habitats can support similar densities of prey species compared with interior habitats, such as fawn‐footed melomys (*Melomys cervinipes*; Avgar et al., 2013; M. Goosem, 2000; Harrington et al., 2001). These roads could also reduce travel costs for feral cats. The expansion and maintenance of road networks within the AWT may allow feral cats to reach areas of the forest that would take longer to colonize naturally (Raiter et al., 2018). The road preferences of feral cats does make it easier to monitor feral cat occurrence and potentially increases the likelihood of encounters, which could be advantageous for control measures. We suggest that opening new trails (for example, the newly proposed Paluma to Wallaman falls or Wangetti trail systems) should be considered carefully, as it could allow feral cats to proliferate within the environment and help them establish populations in previously unaffected rainforest areas.

Our findings highlight the value of targeted monitoring programs for invasive species, particularly along roads. Protected area managers can quickly establish relative abundance estimates and the extent to which feral cats may have penetrated protected areas using this approach. One promising finding of this study was the low number of detections in Paluma, an area that should be highly suitable for feral cats. Paluma could represent a natural refugium for native wildlife reintroductions in the AWT region.

## ACKNOWLEDGEMENTS

We would like to thank the Ecological Society of Australia, Skyrail rainforest foundation, Wet Tropics Management Authority, and James Cook University for providing funding to support the project. The authors would like to acknowledge the traditional owners of the land where this study took place. We also thank the volunteers and students who contributed to fieldwork and processing camera‐trap images throughout the duration of the study.

## AUTHOR CONTRIBUTIONS

**Tom Bruce:** Conceptualization (equal); data curation (lead); formal analysis (lead); funding acquisition (equal); investigation (lead); methodology (lead); project administration (lead); visualization (lead); writing – original draft (lead); writing – review and editing (lead). **Stephen E. Williams:** Conceptualization (equal); formal analysis (supporting); methodology (equal); supervision (equal); writing – original draft (equal); writing – review and editing (equal). **Rajan Amin:** Formal analysis (supporting); supervision (equal); validation (equal); visualization (equal); writing – original draft (equal); writing – review and editing (equal). **Felicity L’Hotellier:** Methodology (supporting); project administration (supporting); resources (equal); writing – original draft (equal); writing – review and editing (equal). **Ben Hirsch:** Conceptualization (equal); formal analysis (supporting); funding acquisition (equal); methodology (equal); project administration (equal); supervision (lead); writing – original draft (equal); writing – review and editing (equal).

## CONFLICT OF INTEREST

The authors have no conflict of interests to declare.

## Supporting information


Appendix S1

## Data Availability

The data that support the findings of this study are openly available in Dryad at https://doi.org/10.5061/dryad.2v6wwpzq4.
