# Supplementary material for: Laying low: Rugged lowland rainforest preferred by feral cats in the Australian Wet Tropics
Source: Ecol Evol. 2022 Jul 13;12(7):e9105. doi: 10.1002/ece3.9105 (PMC9277418; doi:10.1002/ece3.9105)
Supplement: Supplementary file 1 — Appendix S1 [file ECE3-12-e9105-s001.zip › ECE3_9105_Supporting information_final.docx]

**Laying low: Rugged lowland rainforest preferred by feral cats in the Australian Wet Tropics. Supporting material**

Table S1: Broad hypothesis previously suggested to be important for dictating feral cat occupancy and detection probabilities. Each hypothesis summarises several variables tested using occupancy modelling. (r/ λ) indicates the variable was considered to potentially affect occupancy and detection, or (λ) occupancy or detection (*r*) probabilities alone.

|  | Hypothesis | Variables (*r*/ λ) | Method of calculation/data source | References for predictions |
| --- | --- | --- | --- | --- |
| 1 | Increased primary productivity, which is often driven by rainfall, has been linked to increased prey abundance; areas where prey is more abundant, are likely to be more suitable for feral cats. Therefore, we predict that feral cat occupancy would be higher in areas of higher annual precipitation and higher predicted prey biomass. | a) Annual precipitation - (λ)  b) Biomass of prey species - (λ) | a) Rainfall data gathered from accuclim (Storlie et al., 2013)  b) Biomass of prey was calculated using habitat suitability layers generated for mammals likely to constitute prey for feral cats and that had published density estimates. Habitat suitability was modelled by Williams et al., 2010. the formula: ((habitat suitability*Max abundance per kilometer^2^)* body weight) was used to calculate the biomass of each species and then summed. The following species were used; Rusty antechinus (*Antechinus adustus*), Atherton antechinus (*Antechinus godmani*), Atherton Scrubwren (*Sericornis keri*), Northern Red-throated skink (*Carlia rubrigularis*), Chowchilla (*Orthonyx spaldingii*), Grey-headed Robin (*Heteromyias albispecularis*), Musky-rat Kangaroo (*Hypsiprymnodon moschatus*), Red legged pademlon (*Thylogale stigmatica*), Rainforest sunskink (*Lampropholis coggeri*), Fawn-footed Melomys (*Melomys cervinipes*), Long-nosed bandicoot (*Perameles nasuta*), Pale Yellow Robin (*Tregellasia capito*), Bush rat (*Rattus fuscipes*), Cape York rat (*Rattus leucopus*), Basal shade skink (*Saproscincus basiliscus*), Giant white-tailed rat (*Uromys caudimaculatus*), Masked white-tailed rat (*Uromys hadrourus*), Yellow-throated Scrubwren (*Sericornis citreogularis*). | Greenville et al., 2012; Legge et al., 2017; Lock & Wilson, 2017; Williams & Middleton, 2008 |
| 2 | Areas experiencing more anthropogenic disturbance facilitate improved areas for feral cats to hunt and shelter and provide source populations. We predict that areas closer to humans, have lower forest integrity and are increasingly fragmented would have higher feral cat occupancy but lower detection probabilities. The pattern of increased occupancy but lower detection would be due to feral cat populations likely being higher but warier of human persecution. | a) Distance from nearest human population - (*r*/ λ)  b) Forest integrity - (*r*/ λ)  c) Fragmentation - (*r*/ λ) | a) Distance in kilometres from the centre of each raster cell to the nearest human domicile from the QSpatial catalogue (Qspatial, n.d.).  b) Forest integrity index was downloaded from (Grantham et al., 2020) and measures observed and inferred human pressures on forest habitats and how much connectivity has been lost from the broader landscape.  c) Habitat fragmentation was measured using FragScape (Chailloux et al., 2019). We used the Broad Vegetation Habitat layer and road shapefiles from Qspatial, to attain values of effective mesh size, where higher values of effective mesh size indicate less fragmentation (Jaeger, 2000). | Bateman & Fleming, 2012; Dias et al., 2017; Grantham et al., 2020; Krauze-Gryz et al., 2012 |
| 3 | More complex terrain influences opportunities for feral cats to hunt successfully, and elevation drives many species distribution patterns within the Australian Wet Tropics and feral cats in other studies. We predict feral cat occupancy would decrease in more rugged terrain, and the relationship between elevation is inconclusive and is challenging to predict. Detection probability would be higher in topographically complex terrain because the road represents an easier passage through the habitat. | a) Terrain ruggedness index - (*r*/ λ)  b) Elevation - (λ) | a) The Terrain Ruggedness Index (TRI) tool in QGIS was used to calculate TRI. It uses the formula described by(Riley, Deloria, & Elliot, 1999), where each raster pixel contains the difference in elevation from 8 cells surrounding the centre cell.  b) Elevation data was downloaded from Geoscience Australia (GeoscienceAustralia) | Hohnen et al., 2016; McDonald et al., 2020; H. McGregor et al., 2015; Recio et al., 2014; Recio & Seddon, 2013 |
| 4 | Vegetation structural heterogeneity is associated with differing feral cat occupancy values. Where habitats with simpler understories would be predicted to have higher occupancies of feral cats but possibly reduced detection probabilities as they can take alternative paths through the environment.  Primary rainforest habitat understory will be less dense than eucalypt forest and will therefore have lower detection probability, but rainforest habitat likely supports more prey species and, therefore, will have higher occupancy values. | a) Habitat type - (*r*/ λ)  b) Vegetation density in the forest understory - (*r*/ λ) | a) Preclearing and 2019 remanent broad Vegetation Groups of Queensland (BVG) derived from regional ecosystem mapping and were downloaded from Qspatial.  b) Vegetation density in the understory Is the fraction of plant cover at <5m in the habitat, with higher values indicative of more dense understories (Scarth et al., 2019) | H. W. McGregor et al., 2014; Stobo-Wilson et al., 2020 |
| 5 | Increased trapping rates of invasive herbivores are associated with simplifying understory habitat, reducing refugia for native wildlife and improved hunting efficiency for feral cats. The detection probability will decrease as simplified habitat understory will favour feral cats moving away from the road, and occupancy will increase due to improved hunting efficiency, less refuge for native fauna and more effortless movement through the habitat. | a) Trapping rate of invasive herbivores (feral pigs and cows) - (*r*/ λ) | a) Invasive herbivore trapping rate was calculated per camera-trap site by dividing the number of independent photographic events (>60 minutes apart) of cattle and feral pigs by the number of days the camera was operating and then multiplying by 100 (Rovero & Marshall, 2004). | Arrington & Beach, 1999; H. W. McGregor et al., 2014; Mitchell et al., 2007 |
| 6 | Feral cats are positively associated with areas of a habitat that have been burnt as they improve hunting efficiency. We would predict that feral cat occupancy would be higher in more frequently burnt areas due to improvements in hunting efficiency. Detectability would be lower in areas burnt more frequently as feral cats can use alternative routes to travel through the environment due to reduced understory. | a) Fire regime intensity - (*r*/ λ) | a) We used the National Environmental Science Program (NESP) layer of the number of years over the last decade a raster cell has burnt as a proxy for fire regime intensity. | H. W. McGregor et al., 2016 |
| 7 | Physical characteristics of roads may alter the detection probability of species. We predict that narrower roads will have higher detection probabilities. | a) Road width (r) | a) We measured the width of the road where the camera was deployed and at 10m either side of the camera. This resulted in three measurements of road width which were averaged per site. |  |

| **Detection (r)** | **Lambda (λ)** | **nPars** | **AICc** | **ΔAICc** | **AICcWt** |
| --- | --- | --- | --- | --- | --- |
| Camera trap effort | Elevation * Ruggedness | 6 | 909.3 | 0 | 0.72 |
| Camera trap effort | Habitat type | 4 | 911.75 | 2.45 | 0.21 |
| Camera trap effort | Habitat fragmentation * Distance to nearest human population | 6 | 914.34 | 5.04 | 0.06 |
| Camera trap effort | Distance to nearest human population | 4 | 917.72 | 8.42 | 0.01 |
| Camera trap effort | Invasive herbivore trapping rate | 4 | 919.96 | 10.66 | 0 |
| Camera trap effort | Understory vegetation density | 4 | 924.59 | 15.29 | 0 |
| Camera trap effort | Annual precipitation | 4 | 924.9 | 15.6 | 0 |
| Camera trap effort | Fire frequency in the last decade | 3 | 926.17 | 16.87 | 0 |
| Camera trap effort | 1 | 4 | 926.3 | 17 | 0 |
| Camera trap effort | Predicted prey biomass | 4 | 927.86 | 18.56 | 0 |
| 1 | 1 | 5 | 937.86 | 28.56 | 0 |

Table S2: The top models and their rankings when compared to one another taken from the individual hypothesis <4 ΔAIC were considered the best models for explaining patterns of feral cat occupancy

Table S3: MacKenzie and Bailey goodness-of-fit for global Royle-Nichols occupancy model

|  | Cohort | Observed | Expected | Chi-square |
| --- | --- | --- | --- | --- |
| 0 | 1 | 1 | 0.33 | 1.4 |
| 11011111110110100 | 1 | 1 | 0 | 68435.12 |
| 100111001110110000 | 1 | 1 | 0 | 176202.9 |
| 00100110100010010.. | 2 | 1 | 0 | 113750 |
| 0000000000000000... | 3 | 2 | 1.54 | 0.14 |
| 000000000000000.... | 3 | 1 | 0.04 | 20.98 |
| 000000000000010.... | 4 | 3 | 3.51 | 0.07 |
| 000000000000100.... | 4 | 1 | 0.07 | 12.7 |
| 000100000000000.... | 4 | 1 | 0.06 | 13.87 |
| 010110001000000.... | 4 | 1 | 0 | 2883.75 |
| 101001000010000.... | 4 | 1 | 0 | 3750.95 |
| 111101100011000.... | 4 | 1 | 0 | 84328.66 |
| 0000000000......... | 5 | 2 | 1.22 | 0.49 |
| 0100000001......... | 5 | 1 | 0.01 | 86.8 |
| 0100100000......... | 5 | 1 | 0.04 | 24.39 |
| 0101000000......... | 5 | 1 | 0.05 | 16.96 |
| 0101100110......... | 5 | 1 | 0.05 | 17.1 |
| 0101101100......... | 5 | 1 | 0.01 | 76.04 |
| 0101101110......... | 5 | 1 | 0.01 | 84.98 |
| 0110000000......... | 5 | 3 | 0.02 | 481.51 |
| 0110100010......... | 5 | 1 | 0.06 | 14.58 |
| 0111011111......... | 5 | 1 | 0.02 | 62.34 |
| 0111101110......... | 5 | 1 | 0.06 | 14.85 |
| 1011101000......... | 5 | 1 | 0.03 | 27.06 |
| 1100100010......... | 5 | 1 | 0.02 | 62.77 |
| 1110110000......... | 5 | 1 | 0.02 | 62.83 |
| 1111000000......... | 5 | 1 | 0.02 | 41.34 |
| 1111100100......... | 5 | 1 | 0.03 | 37.88 |
| 1111101010......... | 5 | 1 | 0.02 | 54.37 |
| 1111110000......... | 5 | 1 | 0.04 | 26.23 |
| 1111111101......... | 5 | 1 | 0.03 | 27.16 |
| 1111111110......... | 5 | 1 | 0.23 | 2.64 |
| 1111111111......... | 5 | 1 | 0.47 | 0.6 |
| 000000000.......... | 5 | 2 | 1.22 | 0.5 |
| 000000001.......... | 6 | 16 | 15.6 | 0.01 |
| 000000100.......... | 6 | 1 | 0.58 | 0.31 |
| 000010000.......... | 6 | 1 | 0.63 | 0.22 |
| 000011000.......... | 6 | 1 | 0.6 | 0.27 |
| 000100000.......... | 6 | 1 | 0.16 | 4.56 |
| 001000000.......... | 6 | 1 | 0.58 | 0.3 |
| 001010001.......... | 6 | 1 | 0.62 | 0.24 |
| 001011111.......... | 6 | 1 | 0.05 | 19.81 |
| 001100010.......... | 6 | 1 | 0.02 | 49.31 |
| 001101000.......... | 6 | 1 | 0.06 | 16.22 |
| 001110110.......... | 6 | 1 | 0.06 | 14.26 |
| 001111010.......... | 6 | 1 | 0.03 | 35.44 |
| 010000000.......... | 6 | 1 | 0.63 | 0.22 |
| 010011111.......... | 6 | 1 | 0.02 | 47.89 |
| 010110010.......... | 6 | 1 | 0.03 | 28.58 |
| 010111010.......... | 6 | 1 | 0.03 | 34.56 |
| 011000000.......... | 6 | 1 | 0.17 | 4.07 |
| 101001001.......... | 6 | 1 | 0.03 | 37.08 |
| 110010001.......... | 6 | 1 | 0.03 | 34.55 |
| 110100000.......... | 6 | 1 | 0.07 | 13.09 |
| 110111111.......... | 6 | 1 | 0.06 | 14.04 |
| 111000000.......... | 6 | 2 | 0.08 | 48.57 |
| 111011111.......... | 6 | 1 | 0.06 | 14.54 |
| 111101000.......... | 6 | 2 | 0.04 | 100.69 |
| 111101100.......... | 6 | 1 | 0.03 | 29.81 |
| 00000010........... | 7 | 1 | 0 | 377.84 |
| 0100101............ | 8 | 1 | 0 | 1791.62 |
| 00................. | 9 | 2 | 1.64 | 0.08 |
| 01................. | 9 | 1 | 0.22 | 2.78 |
| 0......00000000000. | 10 | 1 | 0.38 | 0.99 |
| 1.................. | 11 | 1 | 0.09 | 9.36 |
|  |  |  |  |  |
| Chi-square statistic = 453413.4 |  |  |  |  |
| Number of bootstrap samples = 10000 | |  |  |  |
| P-value = 0.3941 |  |  |  |  |
|  |  |  |  |  |
| Quantiles of bootstrapped statistics: | |  |  |  |
| 0% | 25% | 50% | 75% | 100% |
| 3100 | 250000 | 380000 | 700000 | 21000000 |
|  |  |  |  |  |
| Estimate of c-hat = 97 |  |  |  |  |


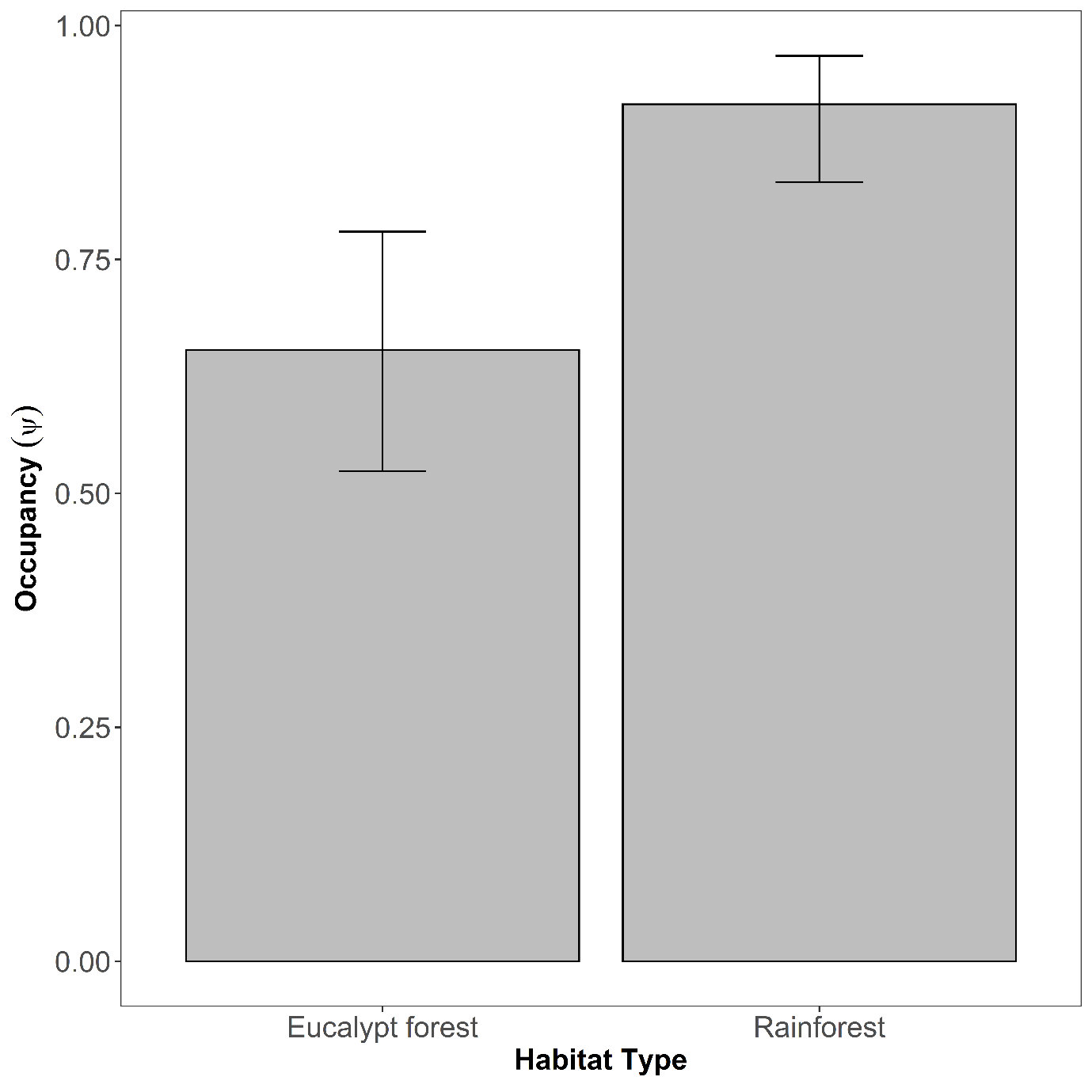


**Figure S1:** Plot showing the effect of habitat type on feral cat site occupancy (Ψ), ±95% Confidence intervals.

**References**

Arrington, D. A., & Beach, W. P. (1999). Effects of rooting by feral hogs SUS SCROFA L. on the structure of a flooplain vegetation assemblage. *The Society of Wetland Scientists*, *19*(3), 535–544.

Bateman, P. W., & Fleming, P. A. (2012). Big city life: Carnivores in urban environments. *Journal of Zoology*, *287*(1), 1–23. https://doi.org/10.1111/j.1469-7998.2011.00887.x

Chailloux, M., Amsallem, J., & Chery, JP. (2019). *FragScape v1.0* (pp. 1–12). https://www.umr-tetis.fr/jdownloads/equipements/FragScape_UserGuide_en.pdf

Dias, R. A., Abrahão, C. R., Micheletti, T., Mangini, P. R., de Oliveira Gasparotto, V. P., de Jesus Pena, H. F., Ferreira, F., Russell, J. C., & Silva, J. C. R. (2017). Prospects for domestic and feral cat management on an inhabited tropical island. *Biological Invasions*, *19*(8), 2339–2353. https://doi.org/10.1007/s10530-017-1446-9

GeoscienceAustralia. (n.d.). *Digital Elevation Data*. Retrieved April 15, 2022, from https://www.ga.gov.au/scientific-topics/national-location-information/digital-elevation-data

Grantham, H. S., Duncan, A., Evans, T. D., Jones, K. R., Beyer, H. L., Schuster, R., Walston, J., Ray, J. C., Robinson, J. G., Callow, M., Clements, T., Costa, H. M., DeGemmis, A., Elsen, P. R., Ervin, J., Franco, P., Goldman, E., Goetz, S., Hansen, A., … Watson, J. E. M. (2020). Anthropogenic modification of forests means only 40% of remaining forests have high ecosystem integrity. *Nature Communications*, *11*(1), 1–10. https://doi.org/10.1038/s41467-020-19493-3

Greenville, A. C., Wardle, G. M., & Dickman, C. R. (2012). Extreme climatic events drive mammal irruptions: Regression analysis of 100-year trends in desert rainfall and temperature. *Ecology and Evolution*, *2*(11), 2645–2658. https://doi.org/10.1002/ece3.377

Hohnen, R., Tuft, K., Mcgregor, H. W., Legge, S., Radford, J., & Johnson, C. N. (2016). *Occupancy of the Invasive Feral Cat Varies with Habitat Complexity*. 1–8. https://doi.org/10.1371/journal.pone.0152520

Jaeger, J. A. G. (2000). Landscape division, splitting index, and effective mesh size: New measures of landscape fragmentation. *Landscape Ecology*, *15*(2), 115–130. https://doi.org/10.1023/A:1008129329289

Krauze-Gryz, D., Gryz, J. B., Goszczyński, J., Chylarecki, P., & Zmihorski, M. (2012). The good, the bad, and the ugly: Space use and intraguild interactions among three opportunistic predators-cat (Felis catus), dog (Canis lupus familiaris), and red fox (Vulpes vulpes)-under human pressure. *Canadian Journal of Zoology*, *90*(12), 1402–1413. https://doi.org/10.1139/cjz-2012-0072

Legge, S. M., Murphy, B. P., McGregor, H. W., Woinarski, J. C. Z., Augusteyn, J., Ballard, G., Baseler, M., Buckmaster, T., Dickman, C. R., Doherty, T. S., Edwards, G., Eyre, T., Fancourt, B. A. A., Ferguson, D., Forsyth, D. M., Geary, W. L. L., Gentle, M., Gillespie, G. R., Greenwood, L., … Zewe, F. (2017). Enumerating a continental-scale threat: How many feral cats are in Australia? *Biological Conservation*, *206*(February), 293–303. https://doi.org/10.1016/j.biocon.2016.11.032

Lock, M., & Wilson, B. A. (2017). Influence of rainfall on population dynamics and survival of a threatened rodent (Pseudomys novaehollandiae) under a drying climate in coastal woodlands of south-eastern Australia. *Australian Journal of Zoology*, *65*(1), 60–70. https://doi.org/10.1071/ZO16084

McDonald, P. J., Stewart, A., Jensen, M. A., & McGregor, H. W. (2020). Topographic complexity potentially mediates cat predation risk for a critically endangered rodent. *Wildlife Research*, *47*(8), 643–648. https://doi.org/10.1071/WR19172

McGregor, H., Legge, S., Jones, M. E., & Johnson, C. N. (2015). Feral cats are better killers in open habitats, revealed by animal-borne video. *PLoS ONE*, *10*(8), 1–12. https://doi.org/10.1371/journal.pone.0133915

McGregor, H. W., Cliff, H. B., & Kanowski, J. (2016). Habitat preference for fire scars by feral cats in Cape York Peninsula, Australia. *Wildlife Research*, *43*(8), 623–633. https://doi.org/10.1071/WR16058

McGregor, H. W., Legge, S., Jones, M. E., & Johnson, C. N. (2014). Landscape management of fire and grazing regimes alters the fine-scale habitat utilisation by feral cats. *PLoS ONE*, *9*(10). https://doi.org/10.1371/journal.pone.0109097

Mitchell, J., Dorney, W., Mayer, R., & McIlroy, J. (2007). Ecological impacts of feral pig diggings in north Queensland rainforests. *Wildlife Research*, *34*(8), 603–608. https://doi.org/10.1071/WR06065

Qspatial. (n.d.). *Queensland Spatial Catalogue : Queensland Government*. Retrieved April 15, 2022, from https://qldspatial.information.qld.gov.au/catalogue/custom/index.page

Recio, M. R., Mathieu, R., Virgós, E., & Seddon, P. J. (2014). Quantifying fine-scale resource selection by introduced feral cats to complement management decision-making in ecologically sensitive areas. *Biological Invasions*, *16*(9), 1915–1927. https://doi.org/10.1007/s10530-013-0635-4

Recio, M. R., & Seddon, P. J. (2013). Understanding determinants of home range behaviour of feral cats as introduced apex predators in insular ecosystems: A spatial approach. *Behavioral Ecology and Sociobiology*, *67*(12), 1971–1981. https://doi.org/10.1007/s00265-013-1605-7

Riley, S. J., DeGloria, S. D., & Elliot, R. (1999). Terrain_Ruggedness_Index.pdf. In *Intermountain Journal of Science* (Vol. 5, pp. 23–27).

Rovero, F., & Marshall, A. R. (2004). Estimating the abundance of forest antelopes by line transect techniques: A case from the udzungwa mountains of tanzania. *Tropical Zoology*, *17*(2), 267–277. https://doi.org/10.1080/03946975.2004.10531208

Scarth, Peter., Armston, J. D., Lucas, Richard., & Butning, Peter. (2019). A structural classification of Australian vegetation using ICESat/GLAS, ALOS PALSAR, and Landsat sensor data. *Remote Sensing*, *11*(2), 147.

Stobo-Wilson, A. M., Stokeld, D., Einoder, L. D., Davies, H. F., Fisher, A., Hill, B. M., Mahney, T., Murphy, B. P., Stevens, A., Woinarski, J. C. Z., Rangers, B., Warddeken, R., & Gillespie, G. R. (2020). Habitat structural complexity explains patterns of feral cat and dingo occurrence in monsoonal Australia. *Diversity and Distributions*, *26*(7), 832–842. https://doi.org/10.1111/ddi.13065

Storlie, C. J., Phillips, B. L., Vanderwal, J. J., & Williams, S. E. (2013). Improved spatial estimates of climate predict patchier species distributions. *Diversity and Distributions*, *19*(9), 1106–1113. https://doi.org/10.1111/ddi.12068

Williams, S. E., & Middleton, J. (2008). Climatic seasonality, resource bottlenecks, and abundance of rainforest birds: Implications for global climate change. *Diversity and Distributions*, *14*(1), 69–77. https://doi.org/10.1111/j.1472-4642.2007.00418.x
