# Supplementary figures and images for: Laying low: Rugged lowland rainforest preferred by feral cats in the Australian Wet Tropics
Source: Ecol Evol. 2022 Jul 13;12(7):e9105. doi: 10.1002/ece3.9105 (PMC9277418; doi:10.1002/ece3.9105)

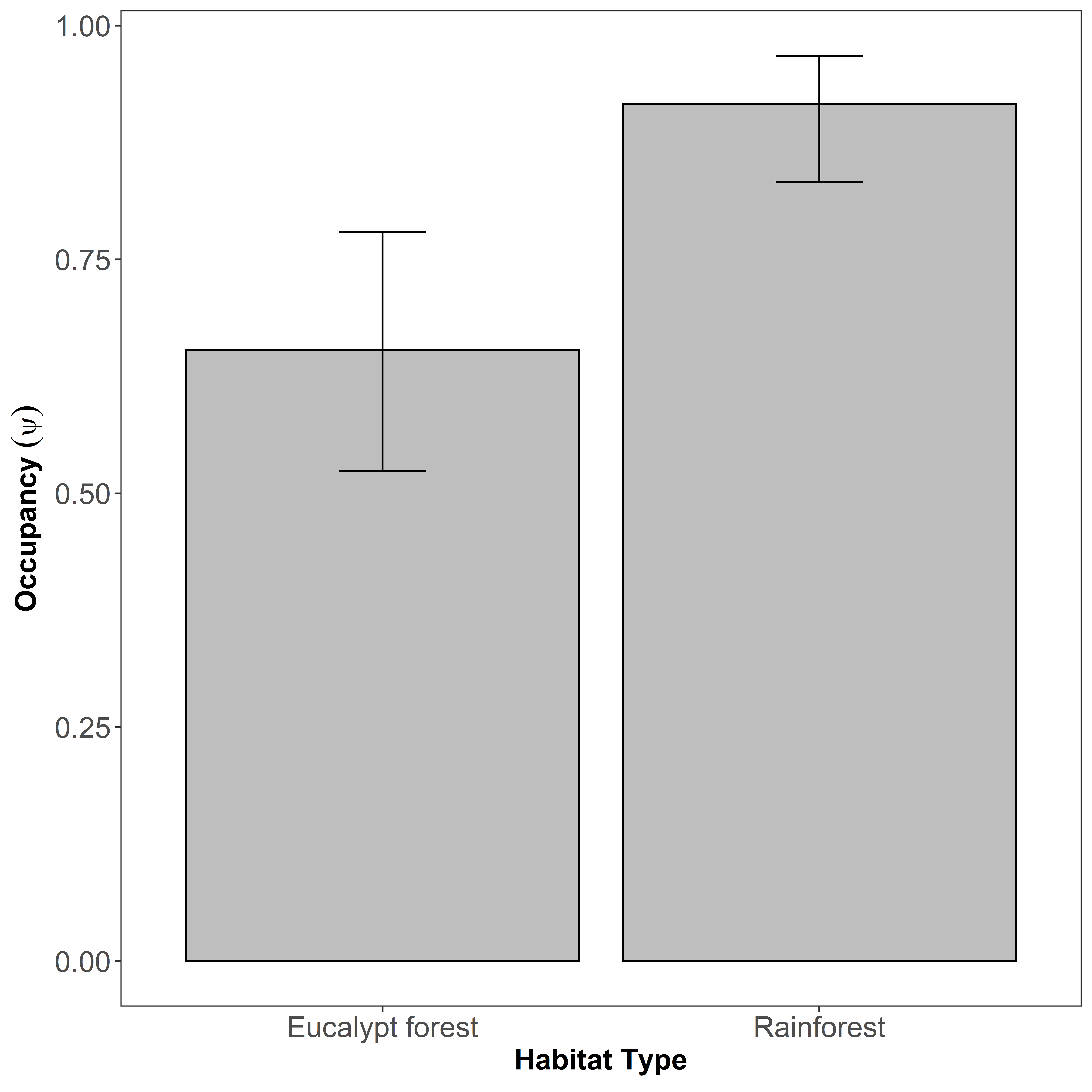

Supplement: Supplementary file 1 — Appendix S1 [file ECE3-12-e9105-s001.zip › ECE3_9105_Habitat.tiff]
